# A Narrative Review on the Multifaceted Roles of Galectins in Host–Pathogen Interactions During *Helicobacter pylori* Infection

**DOI:** 10.3390/ijms26157216

**Published:** 2025-07-25

**Authors:** Bojan Stojanovic, Natasa Zdravkovic, Marko Petrovic, Ivan Jovanovic, Bojana S. Stojanovic, Milica Dimitrijevic Stojanovic, Jelena Nesic, Milan Paunovic, Ivana Milivojcevic Bevc, Nikola Mirkovic, Mladen Pavlovic, Nenad Zornic, Bojan Milosevic, Danijela Tasic-Uros, Jelena Zivic, Goran Colakovic, Aleksandar Cvetkovic

**Affiliations:** 1 Department of Surgery, Faculty of Medical Sciences, University of Kragujevac, 34000 Kragujevac, Serbia; bojan.stojanovic01@gmail.com (B.S.); mpaunovic19691@gmail.com (M.P.); drnikolamirkovic1@gmail.com (N.M.); drmpavlovic1@gmx.com (M.P.); nenadzornic1@gmail.com (N.Z.); drbojanzm1@gmail.com (B.M.); draleksandaracvetkovic@gmail.com (A.C.); 2 Center for Molecular Medicine and Stem Cell Research, Faculty of Medical Sciences, University of Kragujevac, 34000 Kragujevac, Serbia; ivanjovanovic771@gmail.com (I.J.); bojana.stojanovic0@gmail.com (B.S.S.); milicadimitrijevic1@yahoo.om (M.D.S.); 3 Department of Internal Medicine, Faculty of Medical Sciences, University of Kragujevac, 34000 Kragujevac, Serbia; natasasilvester1@gmail.com (N.Z.); jelenazivic1@gmail.com (J.Z.); 4 Department of Pathophysiology, Faculty of Medical Sciences, University of Kragujevac, 34000 Kragujevac, Serbia; 5 Department of Pathology, Faculty of Medical Sciences, University of Kragujevac, 34000 Kragujevac, Serbia; 6 City Medical Emergency Department, 11000 Belgrade, Serbia; ivana.bevc_@yahoo.com (I.M.B.); tasicurosdanijela1@gmail.com (D.T.-U.); colakovicgoran1@gmail.com (G.C.)

**Keywords:** *Helicobacter pylori*, galectins, mucosal immunity, immune evasion, gastric carcinogenesis, β-galactoside-binding lectins, autophagy, host–pathogen interaction, innate immunity, adaptive immunity

## Abstract

*Helicobacter pylori* infection represents one of the most prevalent and persistent bacterial infections worldwide, closely linked to a spectrum of gastroduodenal diseases, including chronic gastritis, peptic ulceration, and gastric cancer. Recent advances have shed light on the critical role of endogenous lectins, particularly galectins, in modulating host–pathogen interactions within the gastric mucosa. Galectins are β-galactoside-binding proteins with highly conserved structures but diverse biological functions, ranging from regulation of innate and adaptive immunity to modulation of cell signaling, apoptosis, and epithelial integrity. This review provides a comprehensive synthesis of current knowledge on the involvement of key galectin family members—especially Galectin-1, -2, -3, -8, and -9—in the context of *H. pylori* infection. Their dual roles in enhancing mucosal defense and facilitating bacterial persistence are examined along with their contributions to immune evasion, inflammation, and gastric carcinogenesis. Understanding the interplay between galectins and *H. pylori* enhances our knowledge of mucosal immunity. This interaction may also reveal potential biomarkers for disease progression and identify novel therapeutic targets. Modulating galectin-mediated pathways could improve outcomes in *H. pylori*-associated diseases.

## 1. Introduction

*Helicobacter pylori* is a highly adapted Gram-negative bacterium that colonizes the human gastric mucosa. The infection is typically acquired in childhood and often persists for decades [1]. Its presence is associated with a broad spectrum of gastroduodenal diseases, ranging from chronic gastritis and peptic ulcer disease to mucosa-associated lymphoid tissue (MALT) lymphoma and gastric adenocarcinoma [2]. While classical studies have emphasized bacterial virulence factors such as cytotoxin-associated gene A (CagA) and vacuolating cytotoxin A (VacA), increasing attention has been directed toward the host’s endogenous modulators of immunity and epithelial integrity, particularly galectins. Galectins are a family of β-galactoside-binding lectins that exhibit diverse immunological and structural functions in both innate and adaptive immune responses [3,4]. Within the gastric mucosa, galectins participate in pathogen recognition, immune cell regulation, epithelial barrier stabilization, and modulation of inflammation and tissue remodeling [4]. Their expression is dynamically regulated during *H. pylori* infection and varies by age, tissue compartment, and inflammatory context. This review explores the multifaceted roles of key galectin family members—Galectin-1 (Gal-1), Galectin-2 (Gal-2), Galectin-3 (Gal-3), Galectin-8 (Gal-8), and Galectin-9 (Gal-9)—in shaping host–pathogen interactions, mediating mucosal defense, and contributing to either containment or persistence of infection as well as their implications in the early stages of gastric carcinogenesis.

In summary, this review provides a comprehensive and integrative overview of the multifaceted roles of galectins in *H. pylori* infection, emphasizing their dual capacity to enhance mucosal immunity while also contributing to bacterial persistence and immune evasion. By consolidating current evidence on galectin-mediated modulation of host–pathogen interactions, the review offers novel insights into potential diagnostic biomarkers and therapeutic targets that could improve clinical management of *H. pylori*-associated diseases.

## 2. Galectins: Conserved Structure, Diverse Functions, and Unique Classification

Galectins represent a distinct group of β-galactoside-binding lectins encoded by the *LGALS* gene family [1]. These proteins are evolutionarily conserved yet functionally diverse, defined by their specific affinity for glycoconjugates containing β-galactoside linkages, such as N-acetyllactosamine [2]. Although 22 galectin genes have been identified across mammalian species, only 16 are known to be expressed in human tissues, with galectins-5, -6, -11, -15, -19, and -20 absent in humans [2].

Despite sharing a conserved structural framework, particularly within their carbohydrate recognition domains (CRDs), galectins exhibit a broad range of biological roles that reflect both evolutionary conservation and specialization [3]. Each galectin contains at least one CRD—a highly homologous motif that governs glycan binding—yet their structural architecture varies, enabling classification into three principal types [4].

**Figure 1:**
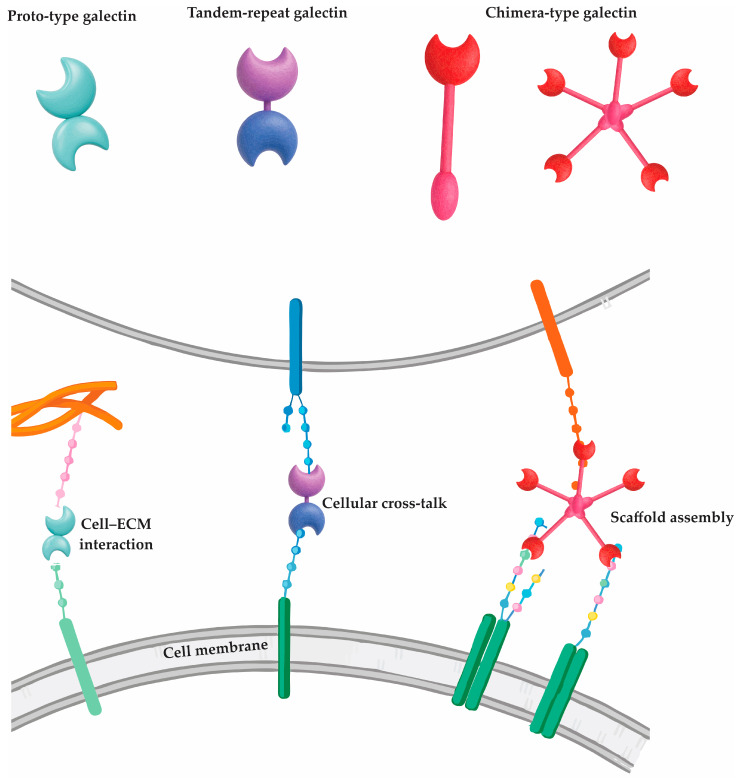
Structural classification of galectins and their modes of glycan-mediated interactions. Galectins (Gal) are grouped into three structural types: proto-type galectins (e.g., Gal-1) contain a single carbohydrate recognition domain (CRD) and typically form non-covalent homodimers; tandem-repeat galectins (e.g., Gal-8) possess two CRDs connected by a linker peptide; and chimera-type galectins (e.g., Gal-3) have one CRD and an N-terminal domain that enables oligomerization into pentamers. The lower panel shows how each galectin type mediates biological functions: proto-type galectins support cell–extracellular matrix (ECM) interactions; tandem-repeat galectins facilitate intercellular cross-talk; and chimera-type galectins form multivalent lattices that organize membrane receptors and assemble signaling platforms.

The proto-type galectins (e.g., Gal-1, -2, -5, -7, -10, -13) possess a single CRD and often form homodimers, primarily through non-covalent interactions [5,6]. These dimeric forms facilitate cross-linking of glycoprotein receptors and participate in diverse signaling cascades [6]. In contrast, tandem-repeat galectins (e.g., Gal-4, -8, -9, -12) harbor two CRDs connected by a short linker peptide, allowing for simultaneous engagement with distinct glycan targets—an attribute that enhances their regulatory flexibility [5]. Galectin-3, the sole chimera-type galectin, is unique in its combination of a single CRD and a non-lectin N-terminal domain that supports oligomerization into pentamers, enhancing its ability to form multivalent glycan lattices [7]. The structural classification of galectins and their corresponding molecular interactions with cell surface and extracellular ligands are illustrated in Figure 1.

Unlike most lectins that are restricted to membrane-associated roles, galectins exhibit a broader subcellular distribution [5]. They localize to various cellular compartments, including the plasma membrane, cytosol, nucleus, endomembrane compartments, extracellular matrix, and even the bloodstream. [5]. This dynamic localization underpins their capacity to regulate processes ranging from intracellular signaling to extracellular matrix remodeling and immune modulation [4].

Furthermore, emerging evidence suggests that galectins can form heterodimers and higher-order hetero-oligomers when co-expressed within the same tissue environment, adding another layer of complexity to their regulatory potential [6]. These interactions may confer context-dependent specificity and functional versatility, reinforcing their significance in both physiological and pathological settings.

### 2.1. Galectins as Multifaceted Regulators of Innate Immune Responses

Galectins, widely expressed in epithelial and immune cells, have emerged as pivotal regulators of innate immunity [4]. Their ability to act both extracellularly through carbohydrate recognition and intracellularly through protein–protein interactions enables them to modulate key aspects of innate immune cell behavior [7,8,9]. The complexity of their involvement stems not only from their structural diversity but also from their dual capacity to either amplify or restrain immune activity depending on the biological context [8,10].

In innate immune signaling, galectins are encountered by immune cells during inflammation, infection, or tissue injury [4]. Gal-3, for instance, is released upon cellular damage and functions as a damage-associated molecular pattern (DAMP), activating innate immune cells [11]. However, many intracellular roles of galectins—especially those independent of glycan binding—have gained increasing recognition [8]. In dendritic cells, both galectin-1 (Gal-1) and Gal-3 regulate cytokine production and T cell-stimulating capacity [12]. Galectin-1 supports immune tolerance, as evidenced in autoimmune disease models, while Gal-3 modulates cytokine output, influencing T helper cell polarization [13,14,15,16,17]. Interestingly, Gal-3 may inhibit or facilitate dendritic cell-mediated T cell activation depending on the microenvironment, suggesting a finely tuned immunoregulatory role [18].

Macrophages, central effectors of innate defense, are likewise under galectin regulation [19]. Galectin-3 enhances NOD-like receptor family pyrin domain containing 3 (NLRP3) inflammasome activity, leading to elevated secretion of interleukin-1 beta (IL-1β) and IL-18, as shown in colitis and hepatobiliary inflammation models [20,21]. Conversely, galectin-9 (Gal-9) attenuates inflammasome activation by promoting the autophagic degradation of NLRP3, highlighting opposing roles within the same pathway [22]. Furthermore, galectins influence macrophage polarization: Gal-3 promotes M2-like, reparative macrophage phenotypes in response to IL-4 and IL-10, while galectin-12 (Gal-12) supports M1-type polarization through the activation of pro-inflammatory signaling cascades [23,24]. These findings underscore the role of galectins in directing macrophage functional specialization.

The phagocytic ability of innate cells is also shaped by intracellular galectins [4]. Galectin-3, localized to the cytosolic side of phagosomes, facilitates actin rearrangement and enhances uptake of opsonized targets [25,26]. Its deficiency impairs both phagocytosis and cytoskeletal organization [27]. Similarly, Gal-9 promotes phagocytic capacity in dendritic cells by supporting actin polymerization, indicating a conserved intracellular role in cytoskeletal regulation [28].

In terms of immune cell trafficking, galectins exhibit context-dependent functions [4]. In skin inflammation such as psoriasis, Gal-3 and galectin-7 (Gal-7) suppress neutrophil recruitment by downregulating pro-inflammatory chemokines and signaling pathways [29,30]. In contrast, Gal-3 promotes neutrophil infiltration in models of airway inflammation and parasitic infection, where it is actively secreted by myeloid cells [31]. Galectin-3 is also involved in eosinophilic recruitment during allergic airway disease, whereas Gal-1 appears to suppress this response [32]. These divergent actions suggest that the tissue environment and type of inflammatory trigger are critical determinants of galectin function.

Overall, galectins orchestrate a diverse set of responses within innate immunity, acting as intracellular regulators, extracellular ligands, and modulators of cytokine networks, phagocytosis, and immune cell migration. Their multifaceted roles offer both therapeutic opportunities and conceptual challenges, particularly as individual galectins can exhibit opposing functions in different contexts. Clarifying the spatiotemporal dynamics of galectin expression and interaction will be essential for harnessing their immunomodulatory potential in infection, autoimmunity, and chronic inflammation.

### 2.2. Galectins as Critical Modulators of T and B Lymphocyte Responses

Galectins are increasingly recognized as key modulators of adaptive immunity, capable of influencing T and B cell fate through both extracellular glycan interactions and intracellular signaling mechanisms [3]. Their regulatory roles span the initiation, expansion, differentiation, and contraction of lymphocyte populations, revealing a dynamic contribution to immune balance and tolerance [4].

Galectins can directly shape T cell activation by binding to glycosylated surface receptors, including cluster of differentiation 2 (CD2), CD7, CD8, CD43, CD45, and the T cell receptor (TCR) [18,33]. This binding alters membrane organization, affects receptor turnover, and modifies the spatial distribution of signaling platforms, ultimately modulating signal strength [33]. Early studies using mannoside acetylglucosaminyltransferase 5 (Mgat5)-deficient mice, which lack the enzyme necessary for producing complex N-glycans, revealed that reduced galectin binding enhances T cell receptor (TCR) signaling and autoimmune susceptibility [34]. These effects were reversible in wild-type cells by disrupting galectin binding with lactose, underscoring the importance of glycan–galectin interactions in controlling immune thresholds [34].

Further insights revealed that Gal-3, whose expression is regulated by IL-10 via the upregulation of Mgat5, can elevate the threshold for T cell activation by enhancing TCR glycosylation [34,35]. This impairs TCR-CD8 co-localization, thereby dampening T cell sensitivity to antigen stimulation [35]. Moreover, Gal-3 functions not only at the membrane but also intracellularly. Upon T cell activation, it relocates to the cytosolic side of the immunological synapse, where it may downregulate the surface expression of key TCR components [18,36]. These intracellular effects may be mediated through interactions with adaptor proteins, such as ALG-2-interacting protein X (ALIX), independent of carbohydrate recognition [37]. Notably, Gal-3 knockout mice exhibit stronger CD8^+^ T cell responses to viral antigens, affirming its role in tempering cytotoxic T cell activation [38].

Galectin-9, like Gal-3, is recruited intracellularly to the immune synapse [39]. It has been linked to enhanced production of pro-inflammatory cytokines, such as IL-17, by activated CD4^+^ T cells. Mice lacking Gal-9 demonstrate impaired T helper 17 (Th17) cell differentiation and reduced immunoglobulin A (IgA) production following oral antigen exposure, suggesting that galectin-9 promotes mucosal immunity by favoring Th17 lineage commitment [40].

Beyond activation, galectins are central to T cell contraction following the resolution of immune responses [4]. Galectin-1 was among the first to be identified as a mediator of T cell apoptosis, with similar apoptotic functions later attributed to galectins -2, -3, -8, and -9 [41,42,43,44]. This pro-apoptotic role is most pronounced in effector T cells, suggesting a selective mechanism to limit immune overactivation. In contrast, intracellular Gal-3 appears to prevent apoptosis by interacting with anti-apoptotic proteins such as B-cell lymphoma 2 (BCL-2), revealing a cell-intrinsic role in T cell survival [45]. In vivo, the deletion of Gal-1, -8, or -9 exacerbates autoimmunity in the experimental autoimmune encephalomyelitis (EAE) model, reinforcing their importance in T cell homeostasis [4]. The regulatory influence of Gal-9 extends to CD8^+^ T cells, as seen in viral infection models where its deficiency results in enhanced cytotoxic responses [46].

The effects of galectins on T cells inevitably impact B cell responses, particularly in T cell-dependent antibody production [4]. Galectin-9 facilitates IgA class switching indirectly by supporting Th17 cell differentiation [40]. In contrast, intracellular Gal-3 impairs B cell maturation into IgA-producing plasma cells [47]. This inhibitory effect was evident in vitro, where B1 cells lacking Gal-3 showed enhanced plasma cell differentiation in response to IL-5 and transforming growth factor-beta 1 (TGF-β1).

Moreover, galectins can directly influence B cell function through surface glycan recognition [48]. Endogenous Gal-9 expressed on naïve B cells regulates receptor clustering and signaling by modulating CD22 and CD45 localization, thereby dampening B cell activation [49]. The disruption of this mechanism through Gal-9 deficiency reduces Src homology region 2 domain-containing phosphatase-1 (SHP1) recruitment, enhances B-cell receptor (BCR) signaling, and accelerates plasma cell differentiation [50]. Similarly, Gal-1 and galectin-8 (Gal-8) affect B cell responses via glycan-mediated interactions, and their inhibition results in diminished plasma cell generation in vitro [51].

### 2.3. Galectins in Host Responses to Bacterial Infections

Galectins play multifaceted roles in shaping the host response to bacterial pathogens, acting both as pattern recognition molecules and as modulators of immune signaling [5]. Through their affinity for β-galactoside-containing glycans, galectins can directly bind to bacterial surface structures such as lipopolysaccharides (LPS) and glycoproteins, influencing bacterial adhesion, immune recognition, and the outcome of infection [3,52,53].

Among Gram-negative bacteria, Gal-3 has been extensively studied for its interaction with LPS, where it can either promote or restrain inflammation depending on the context [54,55,56]. In infections caused by *Salmonella* or *Neisseria meningitidis*, Gal-3 binds to outer membrane components, influencing cytokine production and dampening excessive inflammatory responses. However, it may also facilitate bacterial adhesion and immune evasion by modulating host–pathogen interfaces [56,57].

In contrast, Gal-9 can exert immunosuppressive effects by inducing apoptosis of pro-inflammatory T cell subsets, such as T helper 1 (Th1) and Th17 cells, thereby weakening antibacterial defenses in infections like those caused by *Klebsiella pneumonia* [58]. Similarly, Gal-1 may enhance bacterial invasion by bridging microbial glycoproteins with host integrins, as observed in *Chlamydia trachomatis* and *Porphyromonas gingivalis* infections [59,60].

Intracellularly, galectins also regulate antibacterial autophagy [25]. Galectin-8 typically promotes autophagy by recognizing damaged vacuolar membranes, while Gal-3 can antagonize this process, favoring bacterial persistence [25]. This antagonism is evident in infections with *Listeria monocytogenes* and *Group A Streptococcus*, where galectin-3 suppresses autophagy-associated ubiquitination, thereby limiting bacterial clearance [25].

Interestingly, galectin expression can be modulated by bacterial virulence factors [52]. For instance, during *Yersinia enterocolitica* infection, bacterial effector proteins stimulate Gal-1 expression via mitogen-activated protein kinase (MAPK) signaling, thereby contributing to a tempered mucosal inflammatory response [61].

Together, these findings underscore the context-dependent nature of galectin functions in bacterial infections. While some galectins contribute to host defense by promoting autophagy or enhancing pathogen recognition, others may facilitate immune evasion and microbial persistence. The interplay between galectin subtype, pathogen structure, and host immune status ultimately determines whether their influence is protective or pathogenic. A comparative overview of the immunoregulatory roles of different galectins across innate immunity, adaptive immunity, and bacterial infections is presented in Table 1.

## 3. *Helicobacter pylori* Infection and the Gastric Mucosal Response

*Helicobacter pylori (H. pylori)* is a helical, flagellated Gram-negative bacterium that exhibits remarkable adaptation to the human gastric niche [62]. Colonization typically begins in early childhood and, if untreated, may persist for decades [63]. The bacterium primarily targets the surface of gastric epithelial cells, where it establishes a stable but chronic infection [64]. One of its key survival strategies is the production of urease, an enzyme that catalyzes the hydrolysis of urea into ammonia, thereby buffering gastric acidity and creating a more hospitable microenvironment within the gastric mucus layer [65].

Globally, *H. pylori* infects approximately half of the population, yet clinical outcomes vary widely [66]. While many carriers remain asymptomatic, persistent infection is associated with a continuum of gastroduodenal diseases. These include chronic gastritis, peptic ulcers, and MALT lymphoma as well as systemic manifestations such as iron deficiency anemia and immune thrombocytopenia [66]. Of particular concern is the role of *H. pylori* in gastric carcinogenesis [67]. Long-standing inflammation and epithelial damage promote the progression of atrophic gastritis, intestinal metaplasia, and ultimately gastric adenocarcinoma, leading the International Agency for Research on Cancer to classify *H. pylori* as a Group 1 carcinogen [67,68].

One of the bacterium’s most effective immune evasion mechanisms is molecular mimicry [69]. The outer membrane of *H. pylori* contains LPS with a fucosylated O-antigen that closely resembles Lewis antigens expressed on host epithelial surfaces [69,70]. This mimicry enables the bacterium to camouflage itself and modulate host immune recognition, contributing to its persistence within the gastric mucosa [69].

Recent studies have drawn attention to the role of endogenous lectins—particularly galectins—in shaping the host response to *H. pylori*. Several galectins, including galectin-3, -4, and -9, are expressed in the gastrointestinal tract and are capable of recognizing bacterial glycans such as those found on *H. pylori* LPS [71]. These lectins may contribute to pathogen recognition and immune signaling, yet their function in chronic infection is complex [71]. While galectin binding may support bacterial clearance, it can also influence immune regulation and epithelial responses in ways that inadvertently facilitate persistent colonization [71]. Thus, the interaction between *H. pylori* and host galectins represents a subtle yet important aspect of the host–pathogen interplay within the chronically inflamed gastric mucosa.

### 3.1. Pathogenesis of Helicobacter pylori Infection: Molecular Strategies of Colonization and Immune Evasion

The ability of *Helicobacter pylori* to establish persistent infection within the human gastric mucosa depends on its capacity to overcome mucosal defenses and firmly adhere to the epithelial surface [72]. This initial adhesion is a decisive step in pathogenesis, enabling the bacterium to resist mechanical clearance and initiate downstream virulence mechanisms [73].

The gastric mucosa is equipped with several protective barriers, including surfactant protein D (SP-D) and mucins, which serve as the first line of defense [74]. SP-D, a collagen-containing C-type lectin originally identified in pulmonary surfactant, is also expressed in the gastric lumen, where its concentration increases during *H. pylori*-associated inflammation [74,75]. It binds to bacterial LPS, inhibits motility, and induces aggregation of *H. pylori*, potentially limiting its ability to colonize deeper epithelial layers [74]. In parallel, mucins—highly glycosylated glycoproteins secreted by mucosal epithelial cells—contribute to a biochemical barrier [76]. Particularly, gland mucins rich in O-glycans with terminal α1,4-linked N-acetylglucosamine exhibit direct antimicrobial effects against *H. pylori*, which may explain the confinement of bacteria to the superficial mucous layer [77].

Once in proximity to epithelial cells, *H. pylori* utilizes a repertoire of surface molecules to establish firm adhesion [78]. A key structural feature of its outer membrane is LPS, whose O-antigen chains mimic human blood group antigens, notably Lewis X (LeX), Lewis Y (LeY), Lewis A (LeA), and Lewis B (LeB) [79]. This molecular mimicry serves a dual function—facilitating adhesion and subverting host immune recognition [78]. The expression of these mimicry antigens is phase-variable, allowing *H. pylori* to alternate between different O-antigen structures in successive bacterial generations, a strategy that enhances immune evasion and persistence [80].

Importantly, these glycan structures are not merely passive mimics. They actively participate in adhesion, as demonstrated by the diminished binding capacity of *H. pylori* mutants lacking O-antigen and the ability of LeX-coated particles to replicate bacterial binding patterns on human gastric tissue [81]. The interaction between *H. pylori* surface glycans and host receptors facilitates the delivery of bacterial effector proteins, such as CagA, via the type IV secretion system encoded by the *cag* pathogenicity island [82]. Once translocated into host cells, CagA disrupts epithelial signaling, promotes cellular transformation, and contributes to gastric carcinogenesis [82].

Collectively, *H. pylori* pathogenesis relies on a sophisticated interplay between bacterial glycan mimicry, adhesion molecules, and host epithelial defenses.

### 3.2. Immune Response in Helicobacter pylori Infection: Balancing Inflammation and Persistence

The host immune response to *Helicobacter pylori* is characterized by a complex interplay of pro-inflammatory and immunoregulatory mechanisms that both drive mucosal inflammation and paradoxically support long-term bacterial persistence [83,84]. Central to this response are Th1 and Th17 cells, which mediate protective immunity but are also involved in the development of gastric mucosal pathology [85].

Infection with *H. pylori* activates both Th1 and Th17 pathways, leading to the production of pro-inflammatory cytokines such as interferon-gamma (IFN-γ) and IL-17, which contribute to epithelial inflammation, atrophic changes, and the onset of intestinal metaplasia [85,86,87]. These responses are essential for immune-mediated clearance of the bacterium; indeed, experimental models have demonstrated that the absence of Th1 or Th17 cells results in the failure to eradicate *H. pylori* even after vaccination [85,88]. However, this protective inflammation comes at the cost of tissue damage and long-term gastric alterations.

Interestingly, the magnitude of the Th1 and Th17 responses differs between age groups [89]. In children, *H. pylori*-induced immune activation is generally less intense, resulting in milder histological inflammation compared to adults [89]. This subdued immune profile may facilitate bacterial survival and colonization during early life, a critical period when most persistent infections are established [73,89]. The mechanisms underlying this age-dependent modulation remain incompletely understood but likely involve a combination of host and microbial factors.

*H. pylori* has also evolved virulence strategies that actively suppress protective immune responses [90,91]. Two key effectors—CagA and VacA—play central roles in immune modulation [90,91]. CagA, delivered into host cells via the type IV secretion system, alters intracellular signaling and suppresses co-stimulatory molecule expression, including B7 homolog 2 (B7-H2), thereby dampening Th17 cell development [92]. VacA further skews the immune balance by promoting the differentiation of regulatory T cells (Tregs), which suppress Th1 and Th17 responses and create a tolerogenic environment favorable for persistent colonization [93]. In experimental settings, *H. pylori* strains lacking functional VacA induce more robust inflammatory responses and fail to establish chronic infection, underscoring the immunosuppressive role of this toxin [63].

Beyond T cell regulation, *H. pylori* infection triggers a cascade of innate immune events that amplify inflammation [94]. Activation of nuclear factor kappa-light-chain-enhancer of activated B cells (NF-κB) is a hallmark feature, leading to the production of chemokines such as IL-8, which recruits neutrophils to the site of infection [94]. This influx of immune cells contributes to the oxidative stress and epithelial injury observed in chronic gastritis and promotes a cycle of persistent inflammation [95].

Thus, *H. pylori* orchestrates a finely tuned manipulation of the host immune response: provoking sufficient inflammation to secure a niche within the gastric mucosa while simultaneously dampening specific immune pathways that would otherwise lead to its eradication [95]. Age-related alterations in mucosal immunity significantly influence the host response to *Helicobacter pylori* infection [96]. Immunosenescence, characterized by impaired immune surveillance and chronic low-grade inflammation, contributes to distinct patterns of gastric immunopathology in older adults. Aging is accompanied by gastric atrophy, reduced epithelial regeneration, and changes in cytokine production, collectively reshaping the gastric microenvironment [96]. It is known that Gal-1 expression is increased in chronic gastritis, both in the epithelial and stromal compartments, contributing to the modulation of local immune responses [97]. However, the impact of aging on the expression of galectins within the gastric mucosa, as well as their potential role in age-associated mucosal alterations during *H. pylori* infection, remains unexplored.

**Figure 2:**
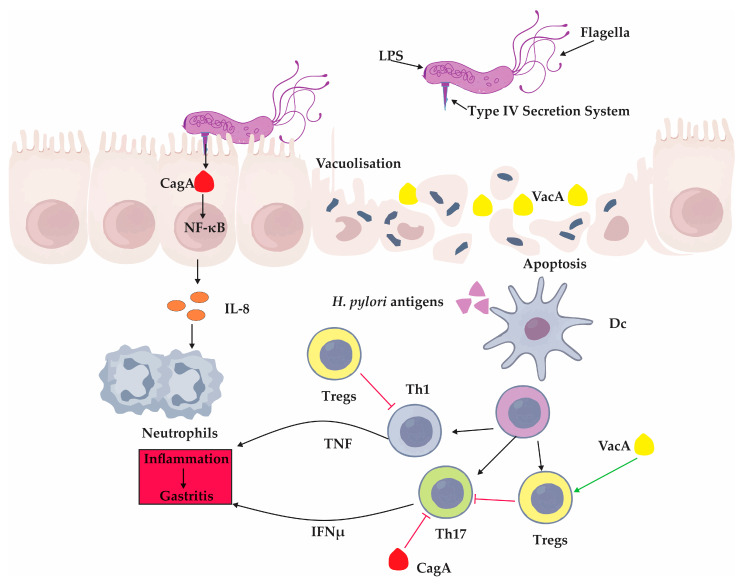
Molecular pathways of *Helicobacter pylori*-induced immune activation, immune evasion, and modulation by galectins within the gastric mucosa. *H. pylori* colonization begins with bacterial motility mediated by flagella, enabling the bacterium to penetrate the gastric mucus layer and adhere to epithelial cells via outer membrane proteins and lipopolysaccharides. The bacterium deploys the type IV secretion system to translocate the CagA effector protein into host epithelial cells, where CagA activates NF-κB signaling. This leads to the upregulation of IL-8 production, driving neutrophil recruitment to the infection site, which exacerbates mucosal inflammation and tissue injury. Simultaneously, VacA toxin enters epithelial cells through endocytosis, where it disrupts mitochondrial membranes, inducing apoptosis of epithelial cells, contributing to mucosal damage. VacA also targets lysosomal membranes, leading to lysosomal dysfunction and oxidative stress, which impairs intracellular pathogen clearance. The immune system responds via Th1 and Th17 polarization, producing IFN-γ and IL-17, respectively, which drive chronic inflammation but are insufficient for bacterial eradication due to immune evasion strategies. *H. pylori* counters these defenses by promoting the expansion of regulatory T cells (Tregs), partly facilitated by VacA- and CagA-mediated suppression of co-stimulatory signals, which collectively inhibit Th1/Th17 responses and promote immune tolerance within the mucosa. Red arrows mean inhibition, green and black arrows mean activation.

The balance between immune activation and immune suppression is pivotal to the bacterium’s long-term survival and the pathogenesis of disease. These key interactions between *H. pylori* virulence factors and the host immune system—including epithelial signaling disruption, cytokine release, and T cell modulation—are summarized in Figure 2.

## 4. Galectin-1: Structure, Immune Regulation, and Its Role in *Helicobacter pylori* Infection

Galectin-1 (Gal-1), a prototypical β-galactoside-binding lectin encoded by the *LGALS1* gene, is a multifunctional molecule that integrates structural, immunological, and context-dependent regulatory functions across physiological and pathological settings. Structurally characterized by its homodimeric β-sandwich configuration and conserved CRDs, Gal-1 engages in diverse cellular processes, including signal transduction, immune regulation, and extracellular matrix interactions. Its ability to modulate both innate and adaptive immune responses, promote immune tolerance, and resolve inflammation underlies its central role in maintaining tissue homeostasis. In the setting of *Helicobacter pylori* infection, Gal-1’s immunosuppressive actions—particularly its attenuation of Th1/Th17-mediated inflammation—have garnered interest for their contribution to bacterial persistence, especially in pediatric populations. The following sections explore the structural biology of Gal-1, its role in host–bacteria interactions, and its emerging significance in *H. pylori*-induced immune modulation and gastritis pathogenesis.

### 4.1. Galectin-1: Structural Features and Multifaceted Immunomodulatory Functions

Galectin-1 is a highly conserved β-galactoside-binding lectin with a molecular weight of approximately 14 kDa, encoded by the *LGALS1* gene [8,98]. Structurally, Gal-1 exists as a homodimer, with each subunit forming a characteristic 135-amino acid β-sandwich fold composed of anti-parallel β-strands [5]. Each monomer harbors a CRD, essential for binding glycosylated ligands in the extracellular environment [8].

Gal-1 is ubiquitously expressed across various tissues and cell types, functioning in both intracellular and extracellular compartments [99]. Intracellularly, Gal-1 has been associated with nuclear processes such as mRNA splicing and signal transduction through weak protein–protein interactions [99]. Extracellularly, Gal-1 can bind to glycosylated receptors on cell surfaces or within the extracellular matrix, where it exerts effects by clustering glycoproteins and modulating cell–cell and cell–matrix adhesion [100,101]. Interestingly, Gal-1 immobilized in the extracellular matrix demonstrates enhanced biological activity compared to its soluble counterpart—capable of inducing T cell apoptosis [100].

Functionally, Gal-1 is involved in a broad spectrum of physiological and pathological processes. It plays key roles in embryonic development, tissue remodeling, muscle differentiation, neuroregeneration, and angiogenesis [102]. In the context of the immune system, Gal-1 modulates inflammation, promotes immune tolerance, and contributes to the resolution of immune responses [103]. These immunomodulatory functions are especially relevant in cancer, where Gal-1 promotes tumor immune escape, and in pregnancy, where it supports maternal–fetal tolerance [103]. Additionally, Gal-1 is implicated in infectious diseases, transplant immunology, and autoimmune disorders, highlighting its context-dependent roles in regulating immune equilibrium [99].

Due to its structural versatility and diverse biological activities, Gal-1 has emerged as a critical node in the network of glycoimmune interactions. Ongoing research continues to uncover its multifaceted contributions to host–pathogen interactions, including in chronic infections such as *Helicobacter pylori*, where its immune-suppressive properties may influence bacterial persistence and disease progression.

### 4.2. Galectin-1 as a Context-Dependent Regulator of Host–Bacterial Interactions

Galectin-1, a β-galactoside-binding lectin widely expressed in mammalian tissues, plays a multifaceted role in bacterial infections, exerting both pro-pathogenic and protective effects depending on the pathogen and host context [99]. This lectin can modulate the course of infection by influencing immune cell activation, cytokine production, and the interactions between bacterial ligands and host glycan structures [99].

In several bacterial models, Gal-1 appears to facilitate microbial persistence. For instance, during *Yersinia enterocolitica* infection, Gal-1 attenuates the host inflammatory response by downregulating the production of interferon-γ, tumor necrosis factor-α, interleukin-17, and nitric oxide [61]. This immunosuppressive effect is further supported by Gal-1’s ability to bind to bacterial virulence factors such as Yersinia outer proteins (Yops), protecting them from proteolytic degradation and thereby maintaining their capacity to subvert host immunity [104].

Similarly, in infections caused by *Tropheryma whipplei* and *Chlamydia trachomatis*, Gal-1 enhances bacterial adherence and entry into host cells [59,105]. This is achieved through a bridging mechanism between bacterial surface glycoproteins and complex N-glycan structures on host membranes, particularly those containing β1,6-branches [59]. Gal-1-mediated enhancement of pathogen–host interaction involves key receptors such as β1 integrins and platelet-derived growth factor receptor beta (PDGFRβ), facilitating internalization and intracellular survival [59].

The role of Gal-1 is not limited to facilitating infection. In *Pseudomonas aeruginosa*-induced corneal keratitis, Gal-1 exhibits tissue-protective properties by mitigating neutrophilic infiltration and suppressing pathogenic Th17 responses, thereby reducing inflammatory damage [106]. This illustrates its dual capacity to limit destructive immune responses while potentially preserving tissue integrity.

Gal-1 also modulates neutrophil behavior, a critical component of early antibacterial defense. Depending on the inflammatory context, it can promote or suppress neutrophil migration and oxidative activity [107,108]. Gal-1 encourages the clearance of apoptotic neutrophils by macrophages, thereby aiding in the resolution of inflammation [109]. However, during acute or sustained inflammation, Gal-1 can inhibit neutrophil adhesion and transmigration, tempering excessive immune infiltration [107].

Experimental models underscore Gal-1’s significance in regulating infection outcomes. Gal-1-deficient mice show heightened inflammatory responses and increased bacterial clearance, particularly in the context of *Yersinia* infection. In contrast, the administration of exogenous Gal-1 can impair microbial eradication, confirming its role in immune suppression under certain conditions [61].

### 4.3. Galectin-1 as a Modulator of Immune Homeostasis in Helicobacter pylori Infection

In the context of *Helicobacter pylori* infection, Gal-1 has emerged as a potent immunomodulatory molecule with distinct regulatory roles in shaping mucosal immunity, particularly in the pediatric population [89]. Its known capacity to suppress pro-inflammatory Th1 and Th17 responses is of particular relevance to the localized immune landscape of *H. pylori*-induced gastritis [110]. Galectin-1 exerts these effects by inducing apoptosis in Th1 and Th17 cells, dampening the secretion of pro-inflammatory mediators such as IL-12, IFN-γ, and tumor necrosis factor-alpha (TNF-α) while simultaneously promoting the anti-inflammatory cytokine IL-10 [110,111]. This pattern of cytokine regulation supports a shift toward immune tolerance and attenuation of chronic inflammation.

Experimental findings in both human and animal models underscore Gal-1’s anti-inflammatory role beyond T cell regulation. Galectin-1 has been shown to inhibit neutrophil infiltration, suppress the release of arachidonic acid from LPS-activated macrophages, and reduce inducible nitric oxide synthase (iNOS) production [112]. These effects collectively contribute to a muted inflammatory response, which is characteristic of pediatric *H. pylori* gastritis and may facilitate persistent bacterial colonization during early life [89].

In clinical observations of *H. pylori*-induced gastritis, the distribution and regulation of Gal-1 within the gastric mucosa reveal a striking regional specificity [113]. In pediatric patients, inflammation tends to be more pronounced in the antrum compared to the corpus [113,114]. Correspondingly, Gal-1 expression is found to be significantly higher in epithelial cells of the corpus, with *H. pylori* infection selectively enhancing its expression in corpus stromal cells but not in the antrum [114]. This differential expression suggests that Gal-1 may mitigate corpus inflammation, contributing to the antral-dominant pattern of gastritis commonly seen in children [113]. In contrast, studies in adults show no infection-induced change in Gal-1 expression in either epithelial or stromal compartments of the antrum, implying an age-dependent regulation of this lectin in response to infection [114]. A summary of the immunomodulatory functions and region-specific expression patterns of Galectin-1 in *Helicobacter pylori* infection is provided in Table 2.

## 5. Galectin-2: Structural Characteristics and Antimicrobial Defense in *Helicobacter pylori* Infection

Galectin-2 (Gal-2) is a 14 kDa β-galactoside-binding lectin, structurally related to Gal-1 and predominantly expressed in gastrointestinal epithelial cells. It contributes to mucosal integrity by stabilizing cell–cell junctions and crosslinking mucins within the gastric mucus layer. In *Helicobacter pylori* infection, Gal-2 plays a direct antimicrobial role by binding to bacterial surface glycoconjugates, promoting aggregation, and inducing bacterial death. These actions highlight Gal-2 as both a barrier-strengthening and bactericidal molecule essential for gastric mucosal defense.

### 5.1. Galectin-2: Structural Insights and Its Role in Gastric Mucosal Integrity

Galectin-2, a proto-type member of the galectin family, shares significant structural homology with Gal-1, with which it has approximately 43% amino acid sequence identity [43,115]. First identified in human hepatoma HepG2 cells, Gal-2 is a 14 kDa protein encoded by the *LGALS2* gene located on chromosome 22, in close proximity—though on the opposite strand—to the *LGALS1* gene encoding Gal-1 [116]. This proximity may suggest a shared evolutionary origin or coordinated regulatory mechanisms.

Functionally, Gal-2 has garnered attention for its immunomodulatory and barrier-enhancing properties within the gastrointestinal tract [117]. Unlike many other galectins, Gal-2 is predominantly expressed in the gastrointestinal epithelium, including the surface mucous and neck cells of the stomach, goblet cells of the small intestine, and brush border of colonic enterocytes [118,119,120]. It is also present in extra-intestinal tissues such as the placenta and cardiovascular system [121,122]. Upon synthesis in the cytoplasm, Gal-2 can be transported to the cell membrane, transported into the nucleus, or secreted extracellularly via non-classical pathways likely involving endosomal vesicles [4].

One of the most notable roles of Gal-2 is its ability to enhance mucosal barrier function. It achieves this by binding to mucin proteins, such as mucin 5AC (MUC5AC), and facilitating their crosslinking within the mucus layer [117,123]. This interaction strengthens the viscoelastic properties of gastric mucus, reinforcing its protective barrier against luminal insults, including acid, enzymes, and microbial pathogens like *Helicobacter pylori* [117,123]. Gal-2 also binds various glycoconjugates, including β1 integrin, ganglioside GM1, mucin 1 (MUC1), β-catenin, and epithelial cadherin (E-cadherin) [124]. Its interaction with β-catenin is particularly relevant in the context of epithelial cell adhesion, as it enhances the stability of β-catenin/E-cadherin complexes at adherens junctions, thus contributing to epithelial integrity [124].

Beyond mechanical reinforcement of the mucosal barrier, Gal-2 has demonstrated immunoregulatory capabilities. It is implicated in the induction of T cell apoptosis and modulation of inflammation, with roles identified in conditions such as colitis and contact dermatitis [43,115,125]. Gal-2 preferentially binds to complex oligosaccharides such as N-acetyllactosamine (LacNAc), with significantly higher affinity than to simple galactose-containing monosaccharides. Structural modifications such as 3-O-sulfation of galactose residues further enhance this binding affinity [126].

### 5.2. Galectin-2 as a Bactericidal Mediator in Gastric Defense Against Helicobacter pylori

Galectin-2 plays a direct antimicrobial role in the gastric mucosa, acting as part of the innate defense system against *H. pylori* [127]. Its protective activity is mediated through specific interactions with β-galactoside-containing glycoconjugates present on the bacterial surface, particularly within LPS structures [127]. Galectin-2 demonstrates a selective affinity for the H type I blood group antigen expressed in the O-antigen portion of *H. pylori* LPS, enabling it to recognize and bind bacterial targets in a pH-dependent manner, especially at pH 6.0, which mimics the local gastric environment [127].

Functionally, Gal-2 exhibits dual antibacterial mechanisms: it induces bacterial aggregation and exerts direct bactericidal effects [68]. The protein forms homodimers via its CRDs, enabling it to crosslink surface-expressed β-galactoside residues on neighboring *H. pylori* cells [68]. This crosslinking promotes aggregation of bacterial cells into dense clumps, a process that is concentration-dependent and competitively inhibited by lactose, a β-galactoside-containing disaccharide, indicating the specificity of Gal-2–glycan interactions [68]. This aggregation not only impairs motility but also correlates with bacterial cell death, particularly in the central regions of the clumps where metabolic disruption and membrane compromise are most pronounced [68].

Microscopic and viability analyses reveal that *H. pylori* cells located at the core of Gal-2-induced aggregates lose viability, whereas peripheral bacteria exhibit partial resistance, suggesting a dose-dependent bactericidal gradient [68]. Although the precise molecular mechanism remains to be fully elucidated, it is hypothesized that Gal-2 may interfere with bacterial membrane integrity and essential metabolic pathways upon glycan binding, ultimately leading to bacterial death.

Interestingly, *H. pylori* infection is associated with the downregulation of Gal-2 expression in gastric tissues, suggesting that the pathogen may evade host defenses by modulating galectin levels [68]. Nevertheless, Gal-2’s ability to agglutinate and kill *H. pylori*—even in the absence of opsonins or immune cell involvement—highlights its role as a non-immune effector molecule capable of reinforcing mucosal barrier function and contributing to the containment of infection near the epithelial surface. The key antimicrobial properties and mechanisms of action of Galectin-2 in *Helicobacter pylori* infection are summarized in Table 3.

## 6. Galectin-3: A Key Regulator of Gastric Mucosal Immunity and Carcinogenic Signaling During *Helicobacter pylori* Infection

Galectin-3 (Gal-3), a chimera-type β-galactoside-binding lectin, plays diverse roles in immunity, epithelial defense, and carcinogenesis. Structurally equipped to form multivalent lattices, Gal-3 is expressed in both immune and epithelial cells, particularly in the gastrointestinal tract. During *Helicobacter pylori* infection, Gal-3 participates in early immune responses, modulates bacterial adhesion, and promotes macrophage activation. Simultaneously, Gal-3 contributes to epithelial survival and proliferative signaling through pathways initiated by bacterial virulence factors such as CagA. This duality positions Gal-3 at the intersection of host defense and gastric oncogenic transformation, reflecting its context-dependent function in shaping infection outcomes.

### 6.1. Galectin-3: A Multifunctional Mediator at the Interface of Immunity and Gastric Mucosal Defense

Galectin-3 is a structurally distinct member of the galectin family, categorized as a chimera-type galectin due to its composite structure comprising a single CRD tethered to a non-lectin N-terminal domain enriched in proline and glycine residues [128,129]. This configuration enables Gal-3 to multimerize upon ligand binding, forming oligomeric lattices that potentiate its biological interactions with glycosylated receptors and extracellular matrix components [129].

Galectin-3 is constitutively expressed in a variety of cell types, with particularly high levels observed in epithelial cells of the gastrointestinal tract and in immune cells, especially activated macrophages [130,131,132]. Within immune compartments, Gal-3 contributes to a broad spectrum of functions, including the enhancement of phagocytosis, prolongation of neutrophil and macrophage survival, and facilitation of neutrophil extravasation during inflammation [31,133]. Its localization in phagocytic cups and bacterium-containing phagosomes underscores its role in intracellular host defense mechanisms, particularly against bacterial pathogens such as *Mycobacterium tuberculosis* [26,134].

Notably, Gal-3 is not confined to intracellular compartments. It is also secreted via non-classical pathways and localizes to the cell membrane and extracellular space, where it engages in additional immunoregulatory activities [5]. One prominent extracellular function of Gal-3 is its ability to bind LPS from various Gram-negative bacteria, thereby modulating the host’s inflammatory response [135]. In this context, Gal-3 has been implicated in dampening excessive responses to LPS, potentially offering protection against endotoxin-induced shock [56].

In the stomach, Gal-3 is selectively expressed by surface mucous epithelial cells and is abundantly secreted into the overlying mucus layer [118,136]. This strategic localization enables it to serve as a first-line defense molecule within the gastric mucosal barrier [136]. Immunohistochemical and immunoblot analyses consistently demonstrate its enrichment at the luminal interface, suggesting a specialized function in mucosal immunity [136]. By interacting with microbial glycoconjugates and stabilizing mucin networks, Gal-3 may contribute to maintaining epithelial integrity while also exerting direct antimicrobial actions.

### 6.2. Galectin-3 in Bacterial Pathogenesis: Dual Roles in Host Defense and Pathogen Survival

Galectin-3 plays a versatile role in bacterial infections by recognizing specific glycans on microbial surfaces [52,129]. In Gram-negative bacteria, it primarily binds to LPS structures—most notably the β-galactoside residues in the outer core and O-antigen [135,137]. Remarkably, even atypical LPS, such as from *Salmonella minnesota*, can be recognized through lipid A, reflecting Gal-3’s structural adaptability [138]. Gram-positive bacteria, although lacking LPS, are not exempt from Gal-3 binding; here, hydrophobic interactions mediate recognition [139].

Functionally, Gal-3 helps regulate the host’s inflammatory response to bacterial LPS, preventing excessive cytokine release and reducing susceptibility to endotoxin-induced shock [56]. However, this regulatory role may come at the cost of impaired bacterial clearance, as seen in Gal-3-deficient models, where stronger Th1 responses and reactive oxygen production promote better pathogen control [11].

Galectin-3 also interferes with intracellular antimicrobial defenses [57]. In *Listeria monocytogenes* infections, it is recruited to disrupted phagosomal membranes, where it suppresses autophagic clearance [25]. Gal-3-deficient macrophages show more efficient bacterial killing, further potentiated by inhibition of autophagy, suggesting Gal-3 subverts this process [25]. Similarly, in *Group A Streptococcus* infection, Gal-3 antagonizes Gal-8-mediated ubiquitin recruitment, impairing bacterial degradation and promoting intracellular persistence, particularly in endothelial cells [140].

Beyond immunity, Gal-3 facilitates bacterial adhesion and invasion [57]. In *Neisseria meningitidis*, it binds both LPS and pilins, enhancing bacterial attachment to immune cells [141]. In ocular and urinary tract infections (*P. aeruginosa*, *P. mirabilis*), Gal-3 acts as a molecular bridge, promoting pathogen–host interactions through glycan recognition [142,143].

### 6.3. Galectin-3 in Helicobacter pylori Infection: A Dual Modulator of Mucosal Defense and Carcinogenic Transformation

The interaction between *Helicobacter pylori* and host gastric epithelium triggers a multifaceted response in which Gal-3 plays a pivotal role. One of the early defensive mechanisms involves Gal-3 secretion by gastric epithelial cells into the mucus layer, where it functions as a physical barrier, entrapping *H. pylori* and limiting its direct contact with epithelial surfaces [11,136]. In murine models deficient in Gal-3, this defensive line is compromised—*H. pylori* penetrates deeper into gastric tissues, and the bacterial load remains significantly elevated both at early and chronic stages of infection [136]. These observations suggest that Gal-3 contributes to mucosal integrity by restricting initial colonization.

The release of Gal-3 appears to be induced by direct bacterial contact, potentially through interactions between bacterial O-antigen side chains and membrane-associated Gal-3 [81]. Intriguingly, the application of recombinant extracellular Gal-3 has been shown to reduce bacterial adhesion, reinforcing its role as a soluble defensive mediator [144]. Beyond preventing attachment, Gal-3 exerts bactericidal effects—its CRD-dependent activity promotes the elimination of phagocytosed *H. pylori* by macrophages [136]. Macrophages lacking Gal-3 exhibit impaired bacterial killing despite unaffected phagocytosis, highlighting lectin’s role in the intracellular handling of the pathogen [136].

However, the role of Gal-3 is not confined to antimicrobial defense. Upon *H. pylori* infection, gastric epithelial and cancer cells upregulate Gal-3 expression, not only increasing its extracellular presence but also accumulating it in the cytoplasm [94]. In this intracellular context, Gal-3 contributes to enhanced proliferation and resistance to apoptosis—traits that align with early tumorigenic transformation [145]. It has also been implicated in conferring resistance to IFN-γ-mediated growth suppression in hyperproliferative gastric cancer cell lines, linking bacterial infection, immune evasion, and cancer progression through Gal-3-mediated pathways [145].

Given the established association between *H. pylori* infection and the development of gastric adenocarcinoma and MALT lymphoma, Gal-3’s upregulation during infection may represent a key endogenous factor contributing to the altered cellular environment that precedes neoplastic transformation [114]. In this context, Gal-3 emerges not only as a modulator of host defense but also as a potential participant in the early molecular events of gastric carcinogenesis.

### 6.4. Galectin-3 Expression and Interaction with H. pylori: A Molecular Axis in Gastric Colonization

Infection of the gastric mucosa by *Helicobacter pylori* prompts a rapid and sustained upregulation of Gal-3, particularly within the gastric stroma and epithelial compartments [114]. This early host response is more than a defensive maneuver—it appears to be intimately linked to the bacterium’s strategy for mucosal colonization [81].

Upon contact with *H. pylori*, gastric epithelial cells, including AGS cell lines, swiftly elevate Gal-3 synthesis and promote its secretion into the extracellular space [81]. This response has been shown to depend on the presence of a functional type IV secretion system, as infections with *cagA* or *cagE* mutants fail to elicit Gal-3 expression [81]. Inhibiting MAPK signaling also suppresses Gal-3 induction, suggesting that translocation of CagA and downstream kinase activation is central to Gal-3 regulation during infection [81].

One of the most intriguing aspects of Gal-3 in *H. pylori* infection is its role as a microbial receptor. The bacterium expresses Lewis-type antigens, particularly polymeric Lewis X motifs, along the O-antigen side chains of its LPS [146,147]. Gal-3 binds with high specificity to these carbohydrate structures, recognizing repeating N-acetyllactosamine motifs, a well-established ligand for its CRD [81]. This binding is inhibited by lactose and specific monoclonal antibodies, confirming the involvement of both the CRD and N-terminal domain in the interaction [81].

Beyond its role in recognizing Lewis antigen-like structures on *H. pylori* LPS, the potential influence of bacterial strain heterogeneity on Gal-3 interactions remains underexplored. Polymorphisms in key virulence factors, particularly CagA and VacA, determine the pathogenic potential of *H. pylori* and shape the host inflammatory response [148]. CagA-positive strains with specific EPIYA motif patterns are linked to stronger oncogenic signaling and more severe gastric pathology [148]. These genetic differences among strains are also associated with variations in the expression of outer membrane structures, including Lewis antigens, which could modulate Gal-3 binding affinity and downstream immune responses. Although direct evidence connecting *H. pylori* strain diversity to functional differences in Gal-3 interactions is still lacking, this represents a significant gap in understanding the pathogen’s immune evasion strategies and warrants future investigation.

The Gal-3–O-antigen binding not only anchors *H. pylori* to the epithelial surface but may also facilitate localized bacterial clustering and enhance mucosal retention. While this binding strengthens adhesion to AGS epithelial cells, it is not the sole mechanism employed by *H. pylori*, indicating a multifactorial adhesion process [81].

### 6.5. Galectin-3 and the Innate Immune Response to H. pylori: Beyond Adhesion and Aggregation

Galectin-3 is increasingly recognized as an active participant in the early immune response to *Helicobacter pylori*, functioning at the crossroads of epithelial signaling, immune cell recruitment, and inflammatory amplification [136]. Its release from infected gastric epithelial cells occurs rapidly and independently of classical secretory pathways, indicating an unconventional, possibly ectocytic, mechanism that allows Gal-3 to act as a first-line alarmin [81,136].

Once in the extracellular milieu, Gal-3 assumes an immunomodulatory role. It promotes chemotaxis of monocytes and neutrophils, helping to mobilize phagocytic cells toward the infected gastric epithelium [11,144]. Furthermore, recombinant Gal-3 has been shown to increase the phagocytic potential of human neutrophils and stimulate the respiratory burst through the activation of nicotinamide adenine dinucleotide phosphate (NADPH) oxidase, thereby enhancing the antimicrobial capacity of the recruited immune cells [149]. These features position Gal-3 not merely as a physical barrier but as an amplifier of innate defense mechanisms.

Importantly, the upregulation of Gal-3 in response to *H. pylori* is tightly linked to bacterial virulence mechanisms. The delivery of the CagA effector protein into host epithelial cells activates the extracellular signal-regulated kinase/mitogen-activated protein kinase (ERK/MAPK) signaling pathway, a cascade known to regulate Gal-3 expression in other cell types undergoing stress or differentiation [81]. Thus, the bacterium exploits its own pathogenic machinery to trigger host cell responses that may initially serve defensive functions but could later contribute to pathological remodeling of the gastric mucosa.

Intracellularly, Gal-3’s functions diverge further. Under certain conditions, post-translational modifications such as phosphorylation can alter its lectin properties and transform it into an anti-apoptotic molecule [150]. This effect, possibly relevant in the setting of persistent *H. pylori* infection, could prolong the survival of epithelial cells harboring oncogenic CagA signaling—creating a microenvironment permissive to early neoplastic transformation [151]. Notably, Gal-3 contains a conserved anti-death motif resembling domains found in the Bcl-2 family, suggesting a convergence of lectin activity and cell survival pathways [152].

### 6.6. Galectin-3 in the Interplay Between H. pylori Infection and Gastric Carcinogenesis

Galectin-3 has emerged as a critical intracellular mediator linking *Helicobacter pylori* infection with oncogenic transformation in gastric epithelial cells [152]. Upon bacterial challenge, Gal-3 undergoes dynamic intracellular redistribution—from a predominantly nuclear localization to cytoplasmic accumulation—facilitated via chromosomal region maintenance 1 (CRM1)-mediated export [94]. This shift is not merely spatial but functional: cytoplasmic Gal-3 contributes to cell survival by modulating NF-κB signaling and enhancing IL-8 secretion, thereby supporting a proinflammatory and pro-proliferative environment [94].

Galectin-3 expression is upregulated in response to CagA translocation via ERK/MAPK activation, linking bacterial virulence to host immune modulation [81]. Elevated Gal-3 levels, in turn, inhibit infection-induced apoptosis and prolong the viability of infected epithelial cells [94]. This anti-apoptotic effect is tightly linked to Gal-3’s capacity to delay cell cycle arrest, a mechanism that may permit the persistence of CagA-injected cells prone to malignant transformation.

Extracellular Gal-3 also plays a complementary role in shaping the tumor-promoting microenvironment. Its presence reduces bacterial adhesion, indirectly suppressing apoptosis, and promotes the recruitment of monocytes to the site of infection—events that contribute to sustained, low-grade inflammation [144]. Such a milieu, marked by chronic immune activation and epithelial hyperproliferation, is a well-established precursor to gastric cancer.

Galectin-3 undergoes notable context-dependent reprogramming that links chronic inflammation to cancer development. In the early stages of *H. pylori* infection, Gal-3 acts as an alarmin and antimicrobial effector by recognizing bacterial LPS, aggregating the pathogen, recruiting neutrophils and monocytes, and amplifying their respiratory burst to limit bacterial load and mucosal invasion [11,81,136,144,149]. However, chronic infection driven by bacterial virulence factors like CagA sustains ERK/MAPK signaling in epithelial cells and upregulates LGALS3 expression [81]. Concurrently, post-translational modifications, such as phosphorylation, transform cytoplasmic Gal-3 into an anti-apoptotic and pro-survival molecule that stabilizes NF-κB, promotes IL-8 production, and fosters epithelial proliferation [94]. This shift also remodels key pathways, including beta-catenin/T cell factor 4 (β-catenin/TCF-4) and protein kinase B/glycogen synthase kinase 3 beta (AKT/GSK-3β); induces expression of invasion-promoting molecules like Fascin-1, protease-activated receptor 1 (PAR-1), and matrix metalloproteinase-1 (MMP-1); and disrupts E-cadherin-mediated adhesion, collectively driving epithelial–mesenchymal transition and invasive behavior [153,154]. Although direct evidence in *H. pylori* gastritis is scarce, Gal-3 likely interacts with other lectin receptors such as sialic acid-binding Siglecs and C-type lectins like dectin-1, which modulate immune responses, including neutrophil apoptosis, dendritic cell cytokine secretion, and NLRP3 inflammasome activation [129,155,156]. Considering the high sialylation of gastric mucins and Gal-3’s capacity to engage non-canonical inflammasome pathways, similar cross-talk in chronic *H. pylori* infection may amplify pro-tumorigenic inflammation and represents an important area for further investigation [4].

A comprehensive overview of Gal-3’s multifaceted roles during *H. pylori* infection and associated carcinogenic transformation is summarized in Table 4.

## 7. Galectin-8: A Dual-Function Sentinel in Intracellular Defense and Inflammatory Signaling During *Helicobacter pylori* Infection

Galectin-8 (Gal-8), a tandem-repeat lectin with distinct glycan-binding domains, plays a pivotal role in cellular responses to membrane damage and bacterial invasion. It acts both as a sensor of lysosomal injury and an inducer of antibacterial autophagy, particularly during infections with *Helicobacter pylori*. Through recognition of exposed intracellular glycans and recruitment of autophagy adaptors, Gal-8 facilitates pathogen clearance. Simultaneously, its extracellular functions contribute to immune activation and cytokine release, positioning Gal-8 at the interface between intracellular immunity and mucosal inflammation.

### 7.1. Structural Characteristics and Biological Functions of Galectin-8

Galectin-8 is a tandem-repeat type β-galactoside-binding lectin encoded by the *LGALS8* gene [157]. It exists in multiple isoforms generated through alternative splicing, with the canonical form comprising two distinct CRDs—N-terminal and C-terminal—linked by a flexible peptide [158]. The two CRDs display different glycan-binding specificities: the N-terminal domain prefers α2,3-sialylated or sulfated β-galactosides, while the C-terminal domain favors branched N-glycans [159]. Full biological activity of Gal-8 depends on the integrity of both CRDs, as inhibition or deletion of either domain abrogates its function [10]. Galectin-8 can multimerize and cross-link glycosylated receptors, thereby influencing a broad range of cellular processes [8].

Biologically, Gal-8 regulates cell adhesion, migration, proliferation, and apoptosis [158]. It mediates integrin clustering and turnover, modulates signaling pathways such as mechanistic target of rapamycin (mTOR) and MAPK, and can act extracellularly by binding to glycoproteins in the extracellular matrix or on the surface of immune and epithelial cells [4,160,161]. Gal-8 also participates in endocytic trafficking and autophagic responses by recognizing intracellular glycans exposed on damaged endosomes and lysosomes [158].

In the immune system, Gal-8 is expressed in lymphoid organs and is rapidly upregulated in response to inflammatory stimuli [162]. In adaptive immunity, it has context-dependent effects on T cells, promoting activation and proliferation at low concentrations and inducing apoptosis or proliferation arrest at higher levels. Gal-8 also supports regulatory T cell differentiation and can suppress effector responses during prolonged immune activation [162]. In B cells, it enhances antigen retention and presentation in germinal centers and promotes IL-10 production [162]. Within the innate immune compartment, Gal-8 activates dendritic cells by upregulating co-stimulatory molecules and cytokine release, promotes neutrophil adhesion and reactive oxygen species production through integrin binding, and modulates natural killer (NK) cell activity by interacting with surface receptors such as bone marrow stromal antigen 2 (BST2) [162]. Collectively, these functions place Gal-8 as an important regulator of immune homeostasis and inflammation.

### 7.2. Galectin-8 in Bacterial Infections: A Key Sentinel in Intracellular Immunity

Galectin-8 has emerged as a key intracellular sensor of membrane damage during bacterial infection [57]. Unlike Gal-3, which may suppress autophagy under certain conditions, Gal-8 actively promotes antibacterial autophagy by recognizing glycans exposed on damaged vacuolar membranes [25,163]. This function is particularly critical in infections caused by cytosol-invading pathogens such as *Listeria monocytogenes* and *Group A Streptococcus* (GAS) [25,140,164].

In epithelial cells infected with GAS, Gal-8 is recruited to damaged vacuoles, where it facilitates the targeting of bacteria for degradation [140]. This recruitment correlates with the enhanced binding of parkin, an E3 ubiquitin ligase that orchestrates ubiquitin-dependent autophagy [140]. Experimental models have shown that the absence of Gal-8 impairs this process, leading to reduced bacterial clearance [140]. Interestingly, in cells lacking both Gal-8 and Gal-3, bacterial replication is even more limited than in Gal-8-deficient cells alone—suggesting a counter-regulatory interaction, where Gal-3 inhibits while Gal-8 promotes autophagic degradation [140].

Further, Gal-8 appears to play a dominant role in epithelial cells, where its expression is higher than in endothelial cells [140]. This cell-type specificity influences the efficiency of autophagic killing of intracellular bacteria and shapes the trajectory of infection. In the absence of Gal-8, bacteria more readily escape immune clearance, emphasizing its function as a molecular bridge between pathogen recognition and intracellular defense pathways [140].

### 7.3. Galectin-8 as a Molecular Sensor of Lysosomal Damage and Modulator of Autophagy in H. pylori Infection

Galectin-8, a tandem-repeat lectin with distinct affinity for O-glycosylated host glycans, emerges as a critical mediator in the cellular response to *Helicobacter pylori* infection [165]. Evidence from in vivo studies in rhesus macaques as well as in vitro human gastric epithelial models indicates that *H. pylori* infection robustly upregulates Gal-8 mRNA expression in gastric tissue [166]. This transcriptional activation correlates with a marked intracellular accumulation of Gal-8 aggregates in infected cells—an effect that reflects underlying lysosomal membrane damage induced by the bacterium [165].

One pivotal mechanism driving Gal-8 aggregation is its interaction with exposed host O-glycans on the cytoplasmic face of disrupted lysosomes [165]. The integrity of lysosomal membranes is compromised during infection, exposing glycan structures typically sequestered within the lysosomal lumen [165]. Gal-8 recognizes these glycans, accumulating around sites of damage and initiating downstream autophagic responses. This glycan-dependent process is supported by data showing that the inhibition of O-glycosylation significantly attenuates Gal-8 aggregation, whereas N-glycosylation blockade has a lesser effect [165].

Interestingly, Gal-8 not only marks damaged organelles but also actively participates in the recruitment of autophagic machinery. Colocalization studies reveal that Gal-8 aggregates are frequently found alongside autophagosomes and the autophagy adaptor protein NDP52, implicating Gal-8 in selective autophagy [165]. The functional depletion of Gal-8 results in diminished autophagic flux during *H. pylori* infection, highlighting a bidirectional regulatory loop whereby Gal-8 facilitates and is sustained by autophagy.

The vacuolating cytotoxin A (VacA) produced by *H. pylori* further compounds this process. As a pore-forming toxin, VacA directly compromises intracellular vesicles, including lysosomes and mitochondria, exacerbating cellular stress [167]. Three mechanisms have been proposed to explain VacA’s role in enhancing Gal-8 accumulation: first, by directly destabilizing lysosomal membranes; second, by promoting mitophagy-related oxidative damage that disrupts lysosomal stability via hydroxyl radical formation; and third, by manipulating autophagic pathways to favor bacterial persistence while still inducing sufficient organelle stress to promote Gal-8 recruitment [165].

Beyond its intracellular functions, Gal-8 may also act as a secreted immunomodulator during infection [4]. Infected epithelial cells demonstrate increased Gal-8 release, which in turn can activate dendritic cells and enhance the production of proinflammatory cytokines [168]. These findings suggest Gal-8 not only orchestrates intracellular defenses but also contributes to the broader inflammatory landscape of *H. pylori*-associated gastritis. The principal mechanisms and functions of Galectin-8 in *Helicobacter pylori* infection are summarized in Table 5.

## 8. Galectin-9: Immune Checkpoint Modulator in Bacterial Infections and *Helicobacter pylori*-Driven Mucosal Tolerance

Galectin-9 (Gal-9) is a tandem-repeat lectin with dual carbohydrate recognition domains that mediate diverse immunoregulatory functions. Its ability to suppress proinflammatory Th1 and Th17 cells while enhancing regulatory T cell differentiation underscores its role in maintaining immune homeostasis during bacterial infections. In the context of *Helicobacter pylori*, Gal-9 expression—particularly in pediatric gastric mucosa—fosters an immunosuppressive environment that facilitates bacterial persistence and reduces tissue damage, thereby contributing to chronic colonization.

### 8.1. Structural Features and Functional Versatility of Galectin-9

Galectin-9 is a member of the tandem-repeat subclass of galectins, distinguished by the presence of two CRDs located at opposite ends of the protein—namely the N-terminal CRD (N-CRD) and the C-terminal CRD (C-CRD) [169]. These domains, each comprising approximately 148–149 amino acids, are connected by a flexible linker whose length varies among isoforms [170]. Despite their structural resemblance, subtle variations in amino acid composition between the two domains endow them with distinct binding specificities, influencing Gal-9’s interactions with cellular receptors and glycan ligands [171].

Comprehensive structural analyses have revealed that the biological functions of Gal-9 are intricately tied to the specialization of its CRDs [171]. The N-CRD is particularly effective in stimulating dendritic cells, while the C-CRD plays a dominant role in modulating T cell fate, including the induction of apoptosis through receptor-mediated signaling [170]. This dual functionality allows Gal-9 to modulate immune responses through both innate and adaptive pathways.

Functionally, Gal-9 exhibits remarkable plasticity across immune contexts [171]. Under conditions of immune hyperactivation, Gal-9 typically exerts a suppressive effect, curbing excessive inflammation and promoting regulatory T cell activity [172]. Conversely, in settings of immune insufficiency, Gal-9 can amplify immune responses, enhancing the function of antigen-presenting cells and facilitating immune activation [172]. This bidirectional immunoregulatory potential underscores Gal-9’s role as a homeostatic checkpoint within the immune system [172].

Given its broad spectrum of activity—from modulating dendritic cell phenotype to regulating T cell viability—Galectin-9 has emerged as a promising therapeutic target in both inflammatory and neoplastic diseases.

### 8.2. Galectin-9 in the Regulation of Host Immunity During Bacterial Infections

Although Gal-9 is primarily known for its role in immune regulation within viral and tumor contexts, emerging evidence has highlighted its functional relevance in bacterial infections, where it modulates both pathogen clearance and host immune tolerance [4]. Its immunomodulatory properties stem largely from its ability to engage the T cell immunoglobulin and mucin domain-containing protein 3 (Tim-3), which is expressed on various immune subsets, particularly Th1 and Th17 lymphocytes [39].

In the setting of *Klebsiella pneumoniae* infection, Gal-9 demonstrates a prominent immunosuppressive function [58]. The experimental administration of exogenous Gal-9 in infected murine models leads to apoptosis of Th1 and Th17 cells, reducing IL-17A production—a cytokine critical for neutrophil recruitment and antimicrobial defense [58]. This suppression also correlates with decreased levels of granulocyte colony-stimulating factor (G-CSF) and macrophage inflammatory protein 2 (MIP-2), two key mediators of granulopoiesis and chemotaxis [58]. As a consequence, bacterial clearance is impaired, and overall survival is diminished in Gal-9-treated animals [58]. These findings suggest that Gal-9 dampens protective immunity during acute bacterial infections by curtailing Th17-driven responses, which may otherwise contribute to efficient pathogen elimination.

Beyond systemic infections, Gal-9 expression has also been implicated at mucosal surfaces [173]. In corneal models of *Pseudomonas aeruginosa* infection, transcriptional profiling of infected tissues revealed an upregulation of Gal-9 expression alongside other galectin family members [174]. Although its precise role in ocular immunity remains under investigation, the coordinated induction of Gal-9 suggests it may participate in localized immune regulation and tissue repair mechanisms during bacterial challenge.

Moreover, Gal-9’s modulatory role is not restricted to direct immune suppression. Through its interactions with Tim-3, Gal-9 may influence the dynamics of antigen-presenting cells and dendritic cell maturation, thereby shaping the early innate immune response and subsequent adaptive immunity [175]. This is particularly relevant in infections characterized by excessive inflammation, where Gal-9-mediated signaling may serve to contain immunopathology, albeit at the cost of compromised bacterial clearance.

### 8.3. Galectin-9 and Immune Modulation in Helicobacter pylori Infection

Galectin-9, a potent immunoregulatory protein, plays a pivotal role in shaping the host immune landscape during *Helicobacter pylori* infection, particularly in the pediatric gastric mucosa [89]. Recent findings indicate that Gal-9 expression is markedly elevated in children with *H. pylori*-positive gastritis, where it appears to orchestrate a regulatory immune profile conducive to bacterial persistence [89].

One of the principal mechanisms through which Gal-9 exerts its effects is via engagement with the checkpoint receptor Tim-3, prominently expressed on Th1 and Th17 cells [42]. This interaction promotes intracellular calcium flux and leads to apoptotic elimination of these proinflammatory T cell subsets [42]. In parallel, Gal-9 enhances the differentiation of inducible regulatory T (Treg) cells by facilitating the co-localization of transforming growth factor-β (TGF-β) receptor I and CD44, a known Treg marker [176]. This dual effect—a contraction of Th1/Th17 responses and expansion of the Treg population—contributes to an immune environment characterized by low-grade inflammation and high tolerance to bacterial antigens.

Importantly, this Gal-9-driven immunological shift is more pronounced in children than in adults [89]. Pediatric gastric mucosa demonstrates increased orkhead box P3-positive (FoxP3^+^) Treg cell infiltration during infection, which correlates with attenuated inflammatory cell recruitment and reduced mucosal damage [177]. This age-dependent regulatory dominance may explain why spontaneous clearance of *H. pylori* is less frequent in children and why colonization often leads to chronic infection rather than acute inflammation.

The spatial distribution of Gal-9 further supports its role in modulating superficial mucosal immunity. Immunohistochemical analyses reveal that Gal-9 is predominantly expressed in the gastric epithelium rather than in the deeper stromal compartments [89]. This localization suggests that Gal-9 primarily influences immune responses in the epithelial barrier zone—precisely where *H. pylori* establishes colonization.

Beyond its immunosuppressive effects, Gal-9 may also contribute directly to bacterial containment. It has been shown to bind LPS structures on Gram-negative bacteria, potentially acting as an opsonin that enhances macrophage recognition and antimicrobial activity [178]. This dual role—immune suppression and bacterial control—may create a finely balanced host–microbial relationship that permits *H. pylori* survival without provoking destructive inflammation.

Interestingly, while Gal-9 expression rises significantly in pediatric infection, similar induction is not observed in adult patients, highlighting a developmental divergence in mucosal immune regulation [114]. This discrepancy reinforces the notion that Gal-9 contributes to the age-specific immune tolerance that underlies persistent *H. pylori* colonization in children [89]. Moreover, inverse correlations between bacterial load and Th17 responses and the observed positive association between Tregs and *H. pylori* density further support Gal-9’s role as a permissive factor for colonization [89]. The immunomodulatory functions and mucosal localization of Galectin-9 in *H. pylori* infection are summarized in Table 6.

To provide an integrative overview of the diverse yet complementary functions of individual galectins discussed in this review, we summarized their structural features, immunomodulatory roles, and specific contributions to *Helicobacter pylori* infection and gastric pathology in Table 7. This comprehensive synthesis underscores the complexity of galectin-mediated host–pathogen interactions and their dual potential to either contain infection or promote bacterial persistence and disease progression.

## 9. Galectins as Emerging Biomarkers and Therapeutic Targets in *Helicobacter pylori*-Associated Pathologies

Galectins hold significant potential as biomarkers for the diagnosis, prognosis, and therapeutic stratification of *H. pylori*-associated diseases, including chronic gastritis, peptic ulcer disease, and gastric cancer. Their differential expression in the gastric mucosa in response to *H. pylori* infection, alongside their regulatory roles in inflammation, immune tolerance, and tissue remodeling, positions them as promising candidates for clinical application.

Galectin-1, with its immunosuppressive profile and region-specific expression in the pediatric gastric mucosa, could serve as a biomarker for early-life infection persistence and for stratifying patients at risk for chronic infection with minimal inflammatory damage [89,110,111]. Similarly, galectin-3 levels, which increase in both epithelial and stromal compartments during infection, correlate with enhanced proliferative signaling and resistance to apoptosis, suggesting its potential as a biomarker for identifying individuals at higher risk of gastric carcinogenesis [110,111,114,145].

Galectin-8 and galectin-9, as modulators of autophagy and immune checkpoint pathways, respectively, present dual opportunities: first, as indicators of the host’s autophagic and immunoregulatory responses to infection and, second, as therapeutic targets to enhance bacterial clearance or modulate pathological inflammation [89,165].

Future clinical studies are warranted to validate galectin-based biomarkers in diverse patient populations and to explore galectin-targeted interventions as adjunctive therapies alongside standard *H. pylori* eradication regimens.

## 10. Clinical Perspectives and Future Directions

Although considerable preclinical evidence underscores the multifaceted roles of galectins in *Helicobacter pylori* infection, their clinical relevance remains insufficiently addressed. Experimental studies have illuminated the dualistic nature of galectin functions in both host defense and bacterial persistence as well as their contribution to mucosal immune modulation and epithelial transformation. However, clinical trials evaluating galectin-targeted therapies in *H. pylori*-associated diseases are currently lacking.

Insights derived from oncology and immunotherapy suggest that pharmacological modulation of galectins, particularly Gal-1 and Gal-3, may have therapeutic potential in conditions where chronic infection contributes to carcinogenesis. In this context, the gastric mucosa represents a compelling model for exploring galectin inhibition strategies aimed at restoring immune competence and mitigating the long-term consequences of persistent infection.

Future research should prioritize translational studies that integrate molecular profiling of galectin expression with clinical parameters in *H. pylori*-infected individuals. Such efforts could clarify the prognostic value of galectin signatures and inform personalized approaches to infection management. Furthermore, exploring galectin inhibition in preclinical models of *H. pylori*-induced gastric pathology may yield novel adjunctive therapies capable of enhancing bacterial clearance while limiting tissue damage and carcinogenic progression.

A comprehensive understanding of the spatial and temporal dynamics of galectin expression during infection as well as their interaction with bacterial virulence factors is critical for advancing this field. These investigations hold promise for the development of innovative therapeutic strategies that target the immunoregulatory axis of galectins, ultimately contributing to more effective control of *H. pylori* infection and its associated diseases.

## 11. Concluding Remarks

This review has synthesized current insights into the roles of galectins in *Helicobacter pylori* infection, highlighting their context-dependent functions in immunity, bacterial persistence, and gastric pathologies. Galectin-1 and Galectin-9 predominantly contribute to immune tolerance by inducing regulatory T cells and suppressing proinflammatory Th1 and Th17 responses, which facilitates chronic colonization, particularly in pediatric mucosa. In contrast, Galectin-2, Galectin-3, and Galectin-8 participate in direct antibacterial defenses through mechanisms such as bacterial aggregation, modulation of phagocytosis, and autophagy induction.

A key theme emerging from these findings is the functional duality of certain galectins, especially Gal-3 and Gal-9, which can protect the host in acute infection yet contribute to immune evasion and pre-neoplastic changes under chronic conditions. This dualism underscores the delicate balance between effective host defense and the risk of pathology driven by prolonged infection and inflammation.

Nonetheless, significant limitations persist in the field. Much of the current evidence is derived from experimental models with variable translational relevance to human disease, particularly in distinguishing pediatric from adult immune responses. Additionally, the spatial and temporal dynamics of galectin expression in vivo remain incompletely characterized. The contribution of galectins to the immunopathology of *H. pylori* also appears to be modulated by bacterial strain heterogeneity and host glycosylation patterns—areas that require further elucidation.

A comprehensive understanding of these aspects is critical to refining our knowledge of galectin-mediated immunity and their implications in gastric diseases. Bridging these gaps will enable the development of targeted therapies that can selectively modulate galectin functions to restore effective immunity without exacerbating tissue damage or carcinogenic risk.

## 12. Materials and Methods

A systematic literature search was conducted using PubMed, Scopus, and Web of Science databases to identify peer-reviewed publications addressing the role of galectins in *Helicobacter pylori* (*H. pylori*) infection. The search encompassed articles published from database inception to 1 June 2024. Medical subject headings (MeSH) and keyword combinations were used, including “galectin”, “*Helicobacter pylori*”, “innate immunity”, “adaptive immunity”, “autophagy”, “gastric mucosa”, “gastric cancer”, “lectin–glycan interaction”, and “bacterial persistence”. Boolean operators (AND/OR) were employed to optimize sensitivity and specificity of the retrieved records.

Eligible publications included original experimental research conducted in vitro, research conducted in vivo (animal models), and clinical studies in humans as well as systematic reviews and meta-analyses focused on the immunomodulatory role of galectins in *H. pylori* pathogenesis. Inclusion criteria required that studies directly explore the functional or mechanistic involvement of galectin family members (especially Gal-1, Gal-2, Gal-3, Gal-8, and Gal-9) in host–pathogen interactions during *H. pylori* infection. Articles addressing downstream effects, such as gastric inflammation, immune evasion, epithelial integrity, autophagic responses, or carcinogenesis, were prioritized. Only English-language publications were considered.

Exclusion criteria encompassed studies that did not include galectin-related mechanisms, lacked relevance to *H. pylori* infection, focused solely on unrelated bacterial pathogens, or presented findings without empirical data (e.g., opinion pieces, commentaries, or conference abstracts). In cases of duplicate data, the most comprehensive or recent version was retained. Special attention was given to studies elucidating the dualistic roles of galectins in enhancing mucosal defense versus facilitating bacterial persistence and their contributions to gastric carcinogenic transformation.

**Figure 3:**
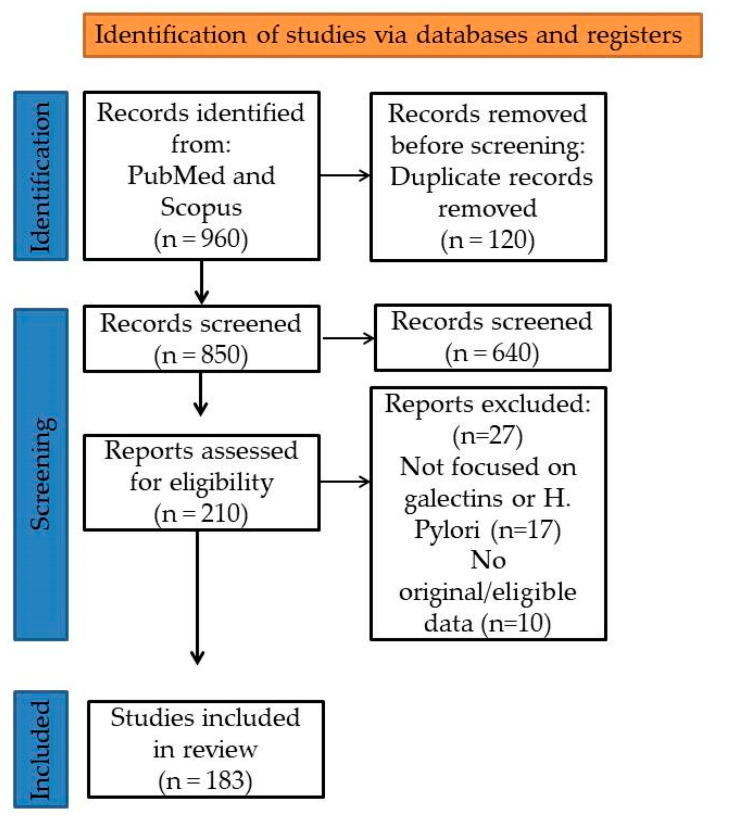
Flowchart of study selection process.

The selected studies were critically appraised and synthesized thematically to reflect the multifaceted roles of galectins in the context of *H. pylori* colonization, immune modulation, and disease progression. The search initially identified 970 records. After removing 120 duplicates, 850 records remained for title and abstract screening. Of these, 640 were excluded due to irrelevance to the research topic. A total of 210 full-text articles were assessed for eligibility. Twenty-seven reports were excluded: 17 did not address galectins or *H. pylori*, and 10 lacked original or eligible data. Ultimately, 175 studies met all inclusion criteria and were incorporated into this review. The study selection process is depicted in Figure 3.

## 13. Conclusions

Galectins have emerged as central regulators in the complex immunobiological landscape of *Helicobacter pylori* infection. Through their context-dependent actions, these lectins contribute to both the containment of bacterial colonization and the maintenance of immune homeostasis while paradoxically enabling immune evasion and chronic persistence. Their involvement spans a wide array of processes—from innate and adaptive immune modulation, epithelial barrier reinforcement, and autophagic regulation to participation in the early events of gastric carcinogenesis. Notably, differences in galectin expression and function across age groups and gastric compartments point to a nuanced and finely tuned regulatory network that evolves with host development and disease progression. Continued investigation into galectin-mediated pathways holds promise for identifying biomarkers of disease risk and progression as well as uncovering novel targets for immunomodulatory or antimicrobial therapies. As our understanding deepens, galectins may not only illuminate the pathogenesis of *H. pylori*-related diseases but also offer broader insights into host–microbe interactions at mucosal surfaces.

## Figures and Tables

**Table 1:** Comparative overview of galectin family members in innate and adaptive immunity and their roles in bacterial infections.

Galectin	Innate Immunity	Adaptive Immunity	Bacterial Infections
Galectin-1	- Induces immune tolerance	- Promotes T cell apoptosis (effector cells)	- Facilitates bacterial invasion via bridging host and bacterial glycoproteins (e.g., *Chlamydia trachomatis*, *P. gingivalis*)
	- Modulates cytokine production in dendritic cells	- Regulates T cell homeostasis (prevents autoimmunity)	- Induced by bacterial effectors via MAPK signaling
	- Suppresses neutrophil recruitment in skin inflammation	- Modulates B cell responses via glycan recognition	
Galectin-3	- Acts as DAMP activating innate immune cells	- Elevates TCR activation threshold by enhancing TCR glycosylation	- Binds LPS and outer membrane of Gram-negative bacteria (*Salmonella*, *Neisseria*)
	- Enhances NLRP3 inflammasome activation (↑ IL-1β, IL-18)	- Downregulates TCR-CD8 co-localization	- Context-dependent inflammation modulation
	- Promotes M2 macrophage polarization	- Prevents T cell apoptosis (via BCL-2 interaction)	- Suppresses autophagy (favors bacterial persistence, e.g., Listeria, *Group A Streptococcus*)
	- Enhances phagocytosis (actin rearrangement)	- Inhibits plasma cell differentiation from B1 B cells	
	- Modulates neutrophil and eosinophil recruitment context-dependently		
Galectin-4	Tandem-repeat structure supports flexible glycan binding (limited specific roles in innate immunity defined)	- Modulates B cell responses (less characterized than Gal-1, -3, -9)	- Limited evidence
Galectin-7	- Suppresses neutrophil recruitment in psoriasis and skin inflammation	- Limited evidence	- Limited evidence
Galectin-8	- Promotes autophagy by recognizing damaged vacuoles	- Induces T cell apoptosis	- Promotes antibacterial autophagy
		- Affects B cell responses via glycan interactions	
Galectin-9	- Attenuates NLRP3 inflammasome via autophagy of NLRP3	- Enhances IL-17 production in CD4^+^ T cells (promotes Th17 differentiation)	- Induces apoptosis of pro-inflammatory Th1/Th17 cells (e.g., in *Klebsiella pneumonia* infection)
	- Promotes M1 macrophage polarization	- Facilitates IgA production	- Immunosuppressive in bacterial infections
	- Enhances phagocytosis in dendritic cells	- Induces apoptosis of effector T cells	
		- Dampens CD8^+^ T cell cytotoxicity	
Galectin-12	- Promotes M1 macrophage polarization (pro-inflammatory)	- Limited evidence	- Limited evidence

**Table 2:** Immunomodulatory functions and spatial expression of Galectin-1 during *Helicobacter pylori* infection, with insights into clinical relevance and potential therapeutic strategies.

Function/Observation	Detail	Clinical Significance/Therapeutic Implication
Suppression of pro-inflammatory T cells	Induces apoptosis of Th1 and Th17 cells	Therapeutic inhibition of Gal-1 could restore Th1/Th17 responses and enhance bacterial clearance in chronic *H. pylori* infection
Cytokine modulation	↓ IL-12, ↓ IFN-γ, ↓ TNF-α; ↑ IL-10	Modulating Gal-1 activity may rebalance cytokine milieu to limit immune suppression
Innate immune modulation	Inhibits neutrophil infiltration and arachidonic acid release from LPS-activated macrophages	Potential target to mitigate immune tolerance that permits persistent colonization
Suppression of iNOS expression	Reduces nitric oxide synthesis in macrophages	Gal-1 inhibition might enhance macrophage bactericidal activity
Role in pediatric gastritis	Promotes tolerance, facilitates persistent colonization, and limits tissue damage	Explains higher *H. pylori* persistence in children; modulation could improve pediatric treatment strategies
Regional expression in gastric mucosa (children)	↑ Gal-1 in corpus epithelium; ↑ in corpus stroma upon infection; no change in antrum	Regional targeting of Gal-1 could prevent corpus-protective, antrum-predominant gastritis patterns
Regional expression in gastric mucosa (adults)	No significant infection-induced change in Gal-1 expression	Gal-1 modulation may still aid in reactivating immune clearance
Contribution to gastritis pattern	May mitigate corpus inflammation, contributing to antral-predominant gastritis in children	Understanding Gal-1 spatial expression could guide precision therapeutics in gastritis management

↑ indicates an increase; ↓ indicates a decrease.

**Table 3:** Antibacterial mechanisms and expression patterns of Galectin-2 in *Helicobacter pylori* infection alongside clinical significance and therapeutic implications.

Function/Observation	Detail	Clinical Significance/Therapeutic Implication
Antibacterial role	Directly kills *H. pylori* and aggregates bacteria via β-galactoside interactions	Gal-2-based therapeutic strategies could reinforce mucosal defenses and limit *H. pylori* colonization
Target specificity	Binds to H type I blood group antigen within *H. pylori* LPS O-antigen	Potential for glycan-targeted therapies enhancing Gal-2-mediated recognition
pH dependency	Binding is optimal at pH 6.0, simulating gastric microenvironment	Pharmacological stabilization of Gal-2 activity at gastric pH could optimize antibacterial effects
Aggregation mechanism	Homodimerizes via CRDs and crosslinks neighboring *H. pylori* cells	Enhancing aggregation properties may restrict bacterial motility and spread
Bactericidal effect	Causes bacterial death in central regions of aggregates via membrane disruption	Gal-2 mimetics could offer direct bactericidal interventions
Concentration dependency	Aggregation and killing are dose-dependent; inhibited by lactose	Local delivery of Gal-2 or analogs might achieve effective concentrations in gastric mucosa
Host expression pattern	Gal-2 expression is downregulated in gastric tissue during *H. pylori* infection	Therapies upregulating Gal-2 may counteract bacterial suppression mechanisms
Immunity-independent function	Acts without opsonins or immune cells; part of innate epithelial defense	Promotes epithelial-autonomous defense; potential adjunctive approach to antibiotic therapy

**Table 4:** Dual role of Galectin-3 in *Helicobacter pylori* infection and gastric carcinogenesis, including clinical significance and potential for therapeutic targeting.

Function/Observation	Detail	Clinical Significance/Therapeutic Implication
Mucosal defense (extracellular)	Secreted by gastric epithelial cells into mucus; entraps *H. pylori* and limits colonization	Targeting Gal-3 may reinforce mucosal defenses to reduce bacterial burden
Effect on bacterial adhesion	Recombinant Gal-3 reduces adhesion of *H. pylori* to epithelial cells	Therapeutic supplementation of recombinant Gal-3 might prevent early colonization
Role in phagocyte function	Enhances killing of phagocytosed *H. pylori* by macrophages	Boosting Gal-3 expression in immune cells could improve intracellular pathogen clearance
Chemotactic activity	Promotes recruitment of monocytes and neutrophils; stimulates neutrophil respiratory burst	Exploiting Gal-3’s chemotactic properties could enhance innate immune cell infiltration to infection sites
Regulation by bacterial factors	Upregulated by *H. pylori* via CagA-dependent ERK/MAPK signaling	Blocking Gal-3 upregulation may mitigate downstream pro-oncogenic signaling
Interaction with bacterial LPS	Binds polymeric Lewis X motifs via CRD; promotes bacterial clustering and retention	Targeting Gal-3–LPS interactions may reduce mucosal bacterial retention and persistence
Intracellular anti-apoptotic activity	Inhibits apoptosis in infected epithelial cells; modulates NF-κB and IL-8 signaling	Gal-3 inhibition may restore apoptosis and reduce hyperproliferation, lowering cancer risk
Cellular localization dynamics	Shifts from nucleus to cytoplasm via CRM1-mediated export after infection	Pharmacologic modulation of Gal-3 localization might control its pro-survival effects
Role in immune evasion and transformation	Promotes epithelial cell survival and hyperproliferation; contributes to tumor microenvironment	Gal-3 could serve as a biomarker for early neoplastic changes and a target for chemopreventive interventions
Link to gastric cancer	Associated with early carcinogenic changes and immune resistance in gastric mucosa	Targeting Gal-3 may prevent progression from chronic gastritis to gastric cancer

**Table 5:** Functional roles of Galectin-8 during *Helicobacter pylori* infection, highlighting intracellular immune defense mechanisms and clinical/therapeutic implications.

Function/Observation	Detail	Clinical Significance/Therapeutic Implication
Expression pattern	Upregulated in gastric tissue during *H. pylori* infection (human and macaque models)	Gal-8 expression could serve as a biomarker for epithelial stress and infection severity
Intracellular accumulation	Aggregates in response to lysosomal membrane damage	Therapeutic enhancement of Gal-8 activity may improve lysosomal integrity and resistance to bacterial persistence
Glycan recognition	Binds exposed O-glycans on damaged lysosomes; O-glycosylation essential for recruitment	Modulating host glycosylation patterns could optimize Gal-8 recruitment and function
Role in selective autophagy	Recruits autophagy adaptor NDP52; colocalizes with autophagosomes	Augmenting Gal-8 function may restore defective autophagy in *H. pylori*-infected cells, reducing bacterial survival
Effect of Gal-8 depletion	Reduces autophagic flux in infected cells	Gal-8 or mimetic therapies could enhance autophagic clearance in chronic *H. pylori* infection
VacA-dependent enhancement	VacA toxin amplifies lysosomal damage and promotes Gal-8 accumulation	Gal-8 upregulation could counteract VacA-mediated cellular injury, limiting pathogen-induced damage
Mechanisms of VacA action	(1) Pore formation in lysosomes, (2) Mitophagy-induced oxidative stress, (3) Autophagy modulation	Understanding VacA-Gal-8 dynamics may inform combinatorial therapies that target both bacterial toxins and host defenses
Extracellular role	Secreted Gal-8 activates dendritic cells and enhances proinflammatory cytokine production	Extracellular Gal-8 could be harnessed to boost mucosal immune activation in settings of immune evasion
Dual function	Intracellular damage sensor and extracellular immune modulator	

**Table 6:** Immunoregulatory functions of Galectin-9 in *Helicobacter pylori* infection, with clinical significance and potential therapeutic implications.

Function/Observation	Detail	Clinical Significance/Therapeutic Implication
Expression pattern	Upregulated in pediatric gastric mucosa during *H. pylori* infection; not significantly induced in adults	Modulation of Gal-9 could enhance immune clearance in children
Cellular localization	Primarily localized in gastric epithelium; limited expression in stromal compartments	Spatial targeting of Gal-9 may enable compartment-specific therapeutic strategies
Interaction with immune checkpoints	Binds Tim-3 receptor on Th1 and Th17 cells → induces apoptosis via calcium signaling	Gal-9/Tim-3 pathway inhibition may restore Th1/Th17 responses to strengthen bacterial clearance
Treg cell induction	Enhances TGF-β receptor I and CD44 co-localization → promotes inducible Treg differentiation	Targeting Gal-9 may rebalance Treg/effector T cell ratios to limit immune suppression
Immune profile modulation	Suppresses Th1/Th17 responses; expands FoxP3+ Tregs → promotes immune tolerance	Therapeutic modulation may prevent excessive tolerance and chronic bacterial persistence
Age-dependent effect	Stronger immunosuppressive effect and Treg dominance in children compared to adults	Justifies developing age-adapted interventions focusing on Gal-9 inhibition in pediatric gastritis
Effect on inflammation	Correlates with reduced mucosal inflammation and limited immune cell recruitment in children	Fine-tuning Gal-9 levels may optimize the balance between inflammation control and effective immunity
Role in bacterial containment	Binds bacterial LPS; may enhance macrophage recognition and antimicrobial activity	Potential for Gal-9-based adjuvants to enhance innate immune recognition while avoiding tissue damage
Clinical implication	Facilitates chronic colonization with minimal tissue damage in pediatric patients	Inhibiting Gal-9 may improve *H. pylori* eradication rates and reduce long-term complications like gastritis or cancer

**Table 7:** Summary of structural features, immunomodulatory functions, and roles of individual galectins in *H. pylori* infection and gastric pathology.

Galectin	Structural Type	Key Immune Functions	Role in *H. pylori* Infection	Effect on Bacterial Persistence	Contribution to Gastric Pathology	References
Galectin-1	Proto-type (homodimer)	Promotes immune tolerance; induces Th1/Th17 apoptosis; suppresses neutrophil infiltration	Suppresses Th1/Th17-driven inflammation; favors immune tolerance in pediatric gastritis	Facilitates bacterial persistence via immunosuppression	Limits tissue damage in pediatric mucosa; potential role in immune evasion	[89,110,111,112,113,114]
Galectin-2	Proto-type (homodimer)	Stabilizes epithelial junctions; crosslinks mucins; induces bacterial aggregation	Directly binds *H. pylori* LPS; promotes aggregation and bacterial death	Contributes to bacterial clearance via aggregation and bactericidal activity	Reinforces mucosal barrier; may counteract infection-induced damage	[68,117,123,127]
Galectin-3	Chimera-type (pentamerization via N-terminal domain)	Acts as DAMP; enhances phagocytosis; modulates inflammasome activation; anti-apoptotic in T cells	Binds *H. pylori* LPS; enhances macrophage bacterial killing; regulates monocyte/neutrophil chemotaxis	Both limits and facilitates persistence: antimicrobial extracellularly but impairs autophagy intracellularly	Promotes epithelial survival and proliferation; implicated in gastric carcinogenesis	[11,81,94,114,136,144,145,146,147,150,151,152]
Galectin-8	Tandem-repeat (two CRDs)	Senses damaged lysosomes; recruits autophagy adaptors (NDP52); stimulates dendritic cells	Marks lysosomal damage induced by *H. pylori*; promotes selective autophagy	Enhances bacterial clearance via autophagy	Contributes to inflammation and tissue remodeling via cytokine induction	[165,166,168]
Galectin-9	Tandem-repeat (two CRDs)	Suppresses Th1/Th17; enhances Treg differentiation; induces T cell apoptosis	Induces immune tolerance; dampens pro-inflammatory responses	Facilitates persistence by limiting Th1/Th17 responses	Facilitates persistence by limiting Th1/Th17 responses	[42,89,177,178]

## Data Availability

As a review article, this manuscript does not contain original research data but offers a comprehensive synthesis and critical evaluation of previously published studies. All referenced data and materials are properly cited and accessible through the cited sources. Therefore, a data availability statement is not applicable.

## Abbreviations

The following abbreviations are used in this manuscript:

ALIX	ALG-2-interacting protein X
B7-H2	B7 homolog 2
BCL-2	B-cell lymphoma 2
BCR	B-cell receptor
BST2	bone marrow stromal antigen 2
C-CRD	C-terminal CRD
CagA	cytotoxin-associated gene A
CD	cluster of differentiation
CRM1	chromosomal region maintenance 1
CRDs	carbohydrate recognition domains
DAMP	damage-associated molecular pattern
E-cadherin	epithelial cadherin
EAE	experimental autoimmune encephalomyelitis
ECM	extracellular matrix
ERK/MAPK	extracellular signal-regulated kinase/mitogen-activated protein kinase
FoxP3^+^	forkhead box P3-positive
Gal	Galectin
Gal-1	Galectin-1
Gal-3	Galectin-3
Gal-7	Galectin-7
Gal-8	Galectin-8
Gal-9	Galectin-9
Gal-12	Galectin-12
GAS	*Group A Streptococcus*
G-CSF	granulocyte colony-stimulating factor
GM1	ganglioside GM1
IFN-γ	interferon-gamma
IgA	immunoglobulin A
IL	interleukin
iNOS	inducible nitric oxide synthase
LacNAc	N-acetyllactosamine
LeA	Lewis A
LeB	Lewis B
LeX	Lewis X
LeY	Lewis Y
LPS	lipopolysaccharides
MAPK	mitogen-activated protein kinase
MALT	mucosa-associated lymphoid tissue
Mgat5	mannoside acetylglucosaminyltransferase 5
MUC1	mucin 1
MUC5AC	mucin 5AC
mTOR	mechanistic target of rapamycin
N-CRD	N-terminal CRD
NADPH	nicotinamide adenine dinucleotide phosphate
NF-κB	nuclear factor kappa-light-chain-enhancer of activated B cells
NK	natural killer
NLRP3	NOD-like receptor family pyrin domain containing 3
PDGFRβ	platelet-derived growth factor receptor beta
SHP1	Src homology region 2 domain-containing phosphatase-1
SP-D	surfactant protein D
TCR	T cell receptor
TGF-β	transforming growth factor-β
TGF-β1	transforming growth factor-beta 1
Th1	T helper 1
Th17	T helper 17
Tim-3	T cell immunoglobulin and mucin domain-containing protein 3
TNF-α	tumor necrosis factor-alpha
Tregs	T cells
VacA	vacuolating cytotoxin A
Yops	Yersinia outer proteins
